# Arabidopsis ADF5 promotes stomatal closure by regulating actin cytoskeleton remodeling in response to ABA and drought stress

**DOI:** 10.1093/jxb/ery385

**Published:** 2018-11-24

**Authors:** Dong Qian, Zhe Zhang, Juanxia He, Pan Zhang, Xiaobin Ou, Tian Li, Lipan Niu, Qiong Nan, Yue Niu, Wenliang He, Lizhe An, Kun Jiang, Yun Xiang

**Affiliations:** 1 MOE Key Laboratory of Cell Activities and Stress Adaptations, School of Life Sciences, Lanzhou University, Lanzhou, China; 2 College of Life Sciences, Zhejiang University, Hangzhou, China

**Keywords:** ABA, ABF/AREBs, actin cytoskeleton remodeling, ADF5, drought, stomatal movement

## Abstract

Stomatal movement plays an essential role in plant responses to drought stress, and the actin cytoskeleton and abscisic acid (ABA) are two important components of this process. Little is known about the mechanism underlying actin cytoskeleton remodeling and the dynamic changes occurring during stomatal movement in response to drought stress/ABA signaling. Actin-depolymerizing factors (ADFs) are conserved actin severing/depolymerizing proteins in eukaryotes, and in angiosperms ADFs have evolved actin-bundling activity. Here, we reveal that the transcriptional expression of neofunctionalized Arabidopsis *ADF5* was induced by drought stress and ABA treatment. Furthermore, we demonstrated that *ADF5* loss-of-function mutations increased water loss from detached leaves, reduced plant survival rates after drought stress, and delayed stomatal closure by regulating actin cytoskeleton remodeling via its F-actin-bundling activity. Biochemical assays revealed that an ABF/AREB transcription factor, DPBF3, could bind to the *ADF5* promoter and activate its transcription via the ABA-responsive element core motif ACGT/C. Taken together, our findings indicate that ADF5 participates in drought stress by regulating stomatal closure, and may also serve as a potential downstream target of the drought stress/ABA signaling pathway via members of the ABF/AREB transcription factors family.

## Teaser

ADF5 promotes stomatal closure by regulating actin filament dynamics, and members of the ABF/AREB transcription factor family may serve as potential upstream regulators of ADF5 in the drought stress/ABA signaling pathway.

## Introduction

Drought stress severely affects plant growth and development, which decreases crop yields and degrades the environment. Terrestrial higher plants have evolved complex mechanisms to adapt to drought stress (Chaves *et al.*, 2002; Reynolds and Tuberosa, 2008; Osakabe *et al.*, 2014; Heyduk *et al.*, 2016). One adaptive strategy involves tight regulation of the opening and closing of stomata (Roelfsema and Hedrich, 2005; Vavasseur and Raghavendra, 2005). The endogenous plant hormone abscisic acid (ABA) plays an important role in the process of stomatal closure. Although many signaling molecules involved in stomatal closure have been identified, the molecular mechanism underlying ABA regulation of stomatal closure is not completely understood (Schroeder *et al.*, 2001; Kim *et al.*, 2010).

Transcriptional regulation plays an important role in the plant’s response to ABA signaling and drought stress. Many transcription factors participate in this process (Shinozaki and Yamaguchi-Shinozaki, 2007). ABA-responsive element binding factor (ABF/AREB) is one the most important transcription factors involved in the ABA/drought response. This transcription factor belongs to a small subfamily of basic leucine zipper (bZIP) proteins (Choi *et al.*, 2000; Uno *et al.*, 2000). Nine *ABF*/*AREB* homologs exist in the Arabidopsis genome (Finkelstein and Lynch, 2000; Lopez-Molina and Chua, 2000). The members of this family all bind the conserved sequence motif (C/T)ACGTGGC, generally known as the ABA-responsive element (ABRE) (Jakoby *et al.*, 2002). Recently, Kim *et al.* (2016) reported that the members of the ABF/AREB family could also bind G-box coupling elements (GCEs), which possess an ACGT/C core motif. Among the ABF/AREB family, ABI5 is induced by ABA and is involved in seed dormancy and germination (Lopez-Molina *et al.*, 2001; Vaistij *et al.*, 2013; Zinsmeister *et al.*, 2016). ABF1, AREB1/ABF2, AREB2/ABF4, and ABF3 are induced by ABA, osmosis, and drought, and are involved in osmotic and drought stress (Choi *et al.*, 2000; Uno *et al.*, 2000; Kang *et al.*, 2002; Kim *et al.*, 2004; Fujita *et al.*, 2005; Furihata *et al.*, 2006; Yoshida *et al.*, 2010; Yoshida *et al.*, 2015). However, the roles of these proteins in the regulation of stomatal movement in response to ABA and/or drought stress are unclear.

Several studies have shown that the actin cytoskeleton is involved in the regulation of stomatal movement. Various disorders can cause dysfunctional opening and closing of stomata, and the conformation of the actin cytoskeleton affects the speed of stomatal opening and closing (Zhao *et al.*, 2011, 2016; Jiang *et al.*, 2012; Li *et al.*, 2014). In addition, there are different actin cytoskeleton rearrangement patterns during stomatal opening and closing. For example, the actin cytoskeleton is composed of well-organized and radially oriented actin filaments when stomata are open, whereas the cytoskeleton is organized into longitudinally oriented, long bundled cables when the stomata are closed (Hwang and Lee, 2001; Zhao *et al.*, 2011). Consistent with these findings, pharmacological experiments have demonstrated that the inhibition of reorganization of actin filaments (F-actin) interrupts stomatal opening and closing (Kim *et al.*, 1995; MacRobbie and Kurup, 2007). However, the direct upstream regulators of the actin cytoskeleton in response to drought/ABA signaling are not well characterized.

The actin cytoskeleton is both highly organized and highly dynamic within plant cells, and its rapid reorganization and turnover are precisely regulated by several actin-binding proteins (ABPs) (Staiger and Blanchoin, 2006). Actin-depolymerizing factors (ADFs) are important and conserved ABPs in eukaryotes, which typically function as key regulators of F-actin dynamics and reorganization via their conserved F-actin severing/depolymerizing activity (Hotulainen *et al.*, 2005; Andrianantoandro and Pollard, 2006). Plant ADFs play important roles in various biological processes, such as pollen tube polar growth, hypocotyl elongation, innate immunity, nematode infection, and stomatal movement (Dong *et al.*, 2001; Clément *et al.*, 2009; Tian *et al.*, 2009; Zheng *et al.*, 2013; Henty-Ridilla *et al.*, 2014; Inada *et al.*, 2016; Zhao *et al.*, 2016; Zhu *et al.*, 2017). The *Arabidopsis thaliana* genome contains genes encoding 11 ADF proteins, which can be divided into four subclasses (subclasses I–IV) (Ruzicka *et al.*, 2007). Interestingly, the biochemical functions of higher plant ADFs have varied throughout evolution; members of subclass III (ADF5 and 9) lost their conserved severing/depolymerizing F-actin activity and instead evolved F-actin bundling activity via key amino acid changes resulting from intron-sliding events (Tholl *et al.,* 2011; Nan *et al.*, 2017). Subclass III ADFs evolved only in flowering plants and may participate in physiological processes unique to plants, such as flowering, double fertilization, and stomatal movement. However, with the exception of the regulation of pollen tube growth by ADF5 (Zhu *et al.*, 2017), the physiological function of these subclass III ADFs, especially during the response to abiotic stress, is not well understood.

In this study, we demonstrated that ADF5 participates in drought stress by regulating stomatal closure via its F-actin-bundling activity. In addition, we found that the up-regulation of *ADF5* expression by ABA partly depended on ABF/AREB transcription factors, and DPBF3 could bind to the *ADF5* promoter and activate transcription via the AREB core motif ACGT/C. Thus, ADF5 may have a potential role in coupling ABA signaling and the actin cytoskeleton in the regulation of stomatal movement.

## Materials and methods

### Plant materials and growth conditions

*Arabidopsis thaliana* Col-0 and *Nicotiana benthamiana* were used in this study. The mutants *adf5* (Salk_018325), *abf1* (Salk_038005), *abf2*/*areb1* (Salk_002984), *abf3*/*dpbf5* (Salk_096965), *abf4*/*areb2* (Salk_069523), *dpbf3*/*areb3* (Salk_061079), and *dpbf4*/*eel* (Salk_021965) (Yoshida *et al.*, 2015) were obtained from the Arabidopsis Biological Resource Center. The primers used to identify homozygous lines are listed in Supplementary Table S1 at *JXB* online. The seedlings were grown on Murashige and Skoog (MS) agar medium (0.8%, w/v) for 5–7 days and then transplanted to soil, where they grew under a 16 h light/8 h dark photoperiod, at 23 °C, 60% relative humidity (RH), and a light intensity of 100 μmol m^–2^ s^–1^, for 3–4 weeks. Similarly, *N. benthamiana* plants were grown in soil under a 16 h light/8 h dark photoperiod, at 28 °C and 60% RH, for 3–4 weeks.

### Water loss assays and drought treatment

To analyze water loss, rosette leaves were detached from 4-week-old plants and placed in a glass culture dish on a laboratory bench (at 23 ± 1 °C temperature and 30–40% RH), and the weight of the detached leaves was then measured every 1 h for 6 h. The experiment was performed three times, with three replicates each time. Water loss was expressed as the percentage of fresh weight (FW) lost. For the drought treatment, seedlings were grown in soil for ~10 days under well-watered conditions, after which water was withheld for 10–15 days. The plant phenotypes in response to the drought stress were characterized and recorded as described previously (Zou *et al.*, 2015).

In addition, in accordance with previous reports (Yoo *et al.*, 2010; De Ollas *et al.*, 2018), water deficit stress was imposed by withholding water from pots (300 ml) that contained 65 g [dry weight DW)] of soilless media and nine plants (3 weeks old) each. The pots were irrigated with water to saturation, allowed to drain, and then weighed to obtain their initial weight, after which they were subjected to drought for different periods. The relative soil water content (SWC) was calculated as:

SWC=(final FW−DW)÷(initial weight−DW)×100

The leaf relative water content (RWC) of fully expanded leaves from 4-week-old plants grown in 300 ml pots at different SWC levels was assessed. First, leaves were removed and immediately weighed to obtain their FW. The leaves were then placed into glass culture dishes filled with distilled water. After 24 h, the leaves were detached and blotted to remove external water, after which they were weighed to obtain their leaf turgid weight (TW). Finally, the leaves were dried to a constant weight at 65 °C and then weighed to obtain their DW. The leaf RWC was calculated as:

RWC=(FW−DW)÷(TW−DW)×100

The experiments were repeated three times.

### Stomatal aperture assays

For ABA-induced stomatal closure, stomatal aperture analysis was performed as described previously (Jiang *et al.*, 2012), with slight modifications. First, the rosette leaves were detached and incubated in stomata-opening buffer [10 mM 2-(*N*-morpholino)ethanesulfonic acid (MES)/KOH with 50 mM KCl, pH 6.15] in a petri dish under constant illumination in a greenhouse in which the temperature was 23 °C and the light intensity was 120 μmol m^–2^ s^–1^. The rosette leaves were then transferred to petri dishes that contained 5 μM ABA for another 3 h. For pharmacological assays, the leaves were pretreated with opening buffer for 2.5 h followed by treatment with different concentrations of Jas (0.75 μM, 1 μM, and 1.25 μM) for 30 min, after which the plants were treated with opening buffer that contained 5 μM ABA and the respective concentrations of Jas (0.75 μM, 1 μM, and 1.25 μM). The abaxial epidermis was imaged by using a Nomarki contrast microscope (Axio Imager Z2, Zeiss, Germany), and stomatal apertures were measured via ImageJ software (http://rsb.info.nih.gov/ij/, National Institutes of Health, USA). The experiment was performed three times, and in each experiment 200 stomata were measured.

### RNA extraction and real-time quantitative PCR analysis

Seedlings were grown on MS agar medium (0.8%, w/v) for 12 days under a 16 h light/8 h dark photoperiod at 22 °C. For the ABA treatment, the 12-day-old seedlings were transplanted into liquid MS medium supplemented with 40 μM ABA under shaking, and allowed to grow under light at 22 °C. For the drought treatment, the 12-day-old seedlings were placed into a glass culture dish on a laboratory bench (at 23 ± 1 °C and 30–40% RH). The total RNA was then extracted from leaves of 12-day-old seedlings treated with ABA or drought at different points by using a MiniBEST Plant RNA Extraction kit (TaKaRa). The total RNA was then reverse-transcribed into cDNA with M-MLV reverse transcriptase (TaKaRa). Real-time quantitative PCR (qRT–PCR) analysis was performed with SYBR Premix Ex Taq (TaKaRa); *UBQ11* was used as an internal control. The primer sequences of the ADFs used in this study have been reported previously (Ruzicka *et al.*, 2007), and the other primers used are listed in Supplementary Table S1.

### Visualization of actin filaments by confocal laser scanning microscopy

To visualize the actin filaments of stomata within guard cells, we used transgenic plants harboring Pro*35S*:*FABD2*-*eGFP*, which is a green fluorescent protein (GFP)-based marker used to reveal actin filaments *in vivo* in Arabidopsis (Wang *et al.*, 2008), in the wild-type (WT) and *adf5* backgrounds. The method used was in accordance with previously described methods (Jiang *et al.*, 2012), with minor modifications. Briefly, detached whole leaves were incubated in opening buffer in a growth chamber for 3 h and subsequently treated with 5 μM ABA or mock buffer for another 2 h. Actin filaments on the abaxial epidermis of the leaves were observed, and all images were captured randomly with a TCS SP8 confocal microscope (Leica) equipped with a ×63/1.4 oil objective.

### Quantification of the FABD2-eGFP relative fluorescence intensity, skewness, and array of actin filaments in guard cells

To determine the relative amount of guard cell actin filaments, the FABD2-eGFP relative fluorescence pixel intensity was measured with ImageJ software in accordance with previously described methods (Zhao *et al.*, 2016). To quantify the extent of guard cell actin filaments, skewness was measured with ImageJ software as previously described (Higaki *et al*., 2010). In addition, actin filament arrays were measured in accordance with the methods of Zhao *et al.* (2011) and Jiang *et al.* (2012).

### Chromatin immunoprecipitation assays

Seedlings were grown on MS agar medium (0.8%, w/v) for 5–7 days. They were transplanted to soil and grown under a 16 h light/8 h dark photoperiod at 23 °C and 60% RH for 3–4 weeks and then treated with 40 μM ABA for 6 h, after which the leaves of the seedlings were harvested. Chromatin immunoprecipitation (ChIP) assays were subsequently performed on Arabidopsis seedlings expressing *35S*:*DPBF3*-*HA* and WT seedlings lacking a hemagglutinin (HA)-tag (as a control), in accordance with previously described methods (Ni *et al.*, 2009). qRT–PCR was used to analyze the enriched DNA fragments in conjunction with the primers listed in Supplementary Table S1.

### Yeast one-hybrid assays

Yeast one-hybrid (Y1H) assays were carried out in accordance with the protocol described in the manual of the Matchmaker Yeast One-Hybrid Y1HGold System (Clontech). Various truncated promoters of *ADF5* were amplified by PCR and cloned into a pAbAi vector. The vector was subsequently linearized and introduced into the yeast strain Y1HGold, yielding a bait-reporter strain. The full-length coding DNA sequence of *DPBF3* was amplified and then cloned into a pGADT7 (Clontech) prey vector, which was then transfected into the above-mentioned bait-reporter yeast strain. Aureobasidin A (Clontech) was used as a drug-selectable marker for yeast.

### Transcriptional activation assays

A *N. benthamiana* transient assay system was used to determine how ABF/AREBs activate *ADF5* expression. The *ADF5* promoters and various truncations and mutations were each cloned into a pGWB235-LUC vector to generate reporter constructs. Each reporter construct was then co-transformed with *35S*:*ABF*/*AREB*s into *N. benthamiana* leaves for transcriptional activity assays. Firefly luciferase was assayed via luciferin (Promega) and captured by a Lumazone CA1300B camera (Photometrics).

### Statistical analysis

Statistical analysis to test the data normality of continuous variables was performed using SPSS version 16.0 (IBM). Data are presented as the means ±SEs or ±SDs based on three independent biological replicates. Two-tailed Student’s *t*-tests were performed to determine group differences. The threshold for significance was set at *P*≤0.05.

## Results

### Expression of *ADF5* was up-regulated in response to ABA and drought

**Fig. 1.**
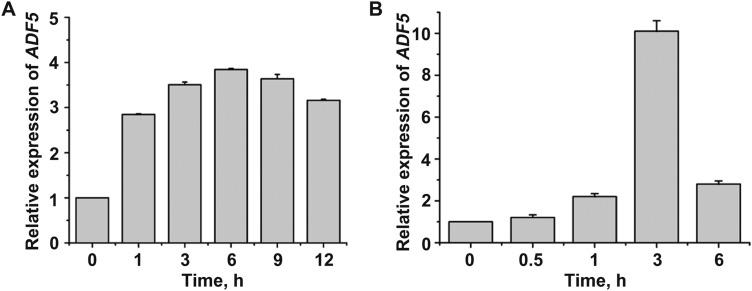
Results of qRT–PCR analysis showing that *ADF5* expression is induced by (A) ABA and (B) drought conditions. Twelve-day-old Arabidopsis seedlings were treated with 40 μM ABA or desiccation for different durations, and the leaves were detached for RNA extraction. *UBQ11* was used as an internal standard. Data presented are the mean ±SD of three independent biological replicates.

Until now, it has been unclear whether and how the subclass III ADFs (ADF5 and ADF9) participate in the response to abiotic stress. We first analyzed the microarray data from AtGenExpress (http://jsp.weigelworld.org/expviz/expviz.jsp; Supplementary Fig. S1) and found that ABA treatments induced the expression of *ADF5* but not *ADF9*. To further confirm these microarray data, we treated 12-day-old WT seedlings with 40 μM exogenous ABA and then performed qRT–PCR. The qRT–PCR analysis indicated that transcriptional expression of *ADF5* was substantially induced by ABA treatments (Fig. 1A) and that the *ADF5* expression level increased but then decreased, which is consistent with the microarray data (Supplementary Fig. S1). Furthermore, we treated 12-day-old WT seedlings by exposing them to drought conditions, and the results were similar to those in response to the ABA treatment (Fig. 1B). Together, these results indicate that ADF5 may participate in the ABA/drought signaling pathway.

### *ADF5* loss of function increases plant sensitivity to drought stress

**Fig. 2.**
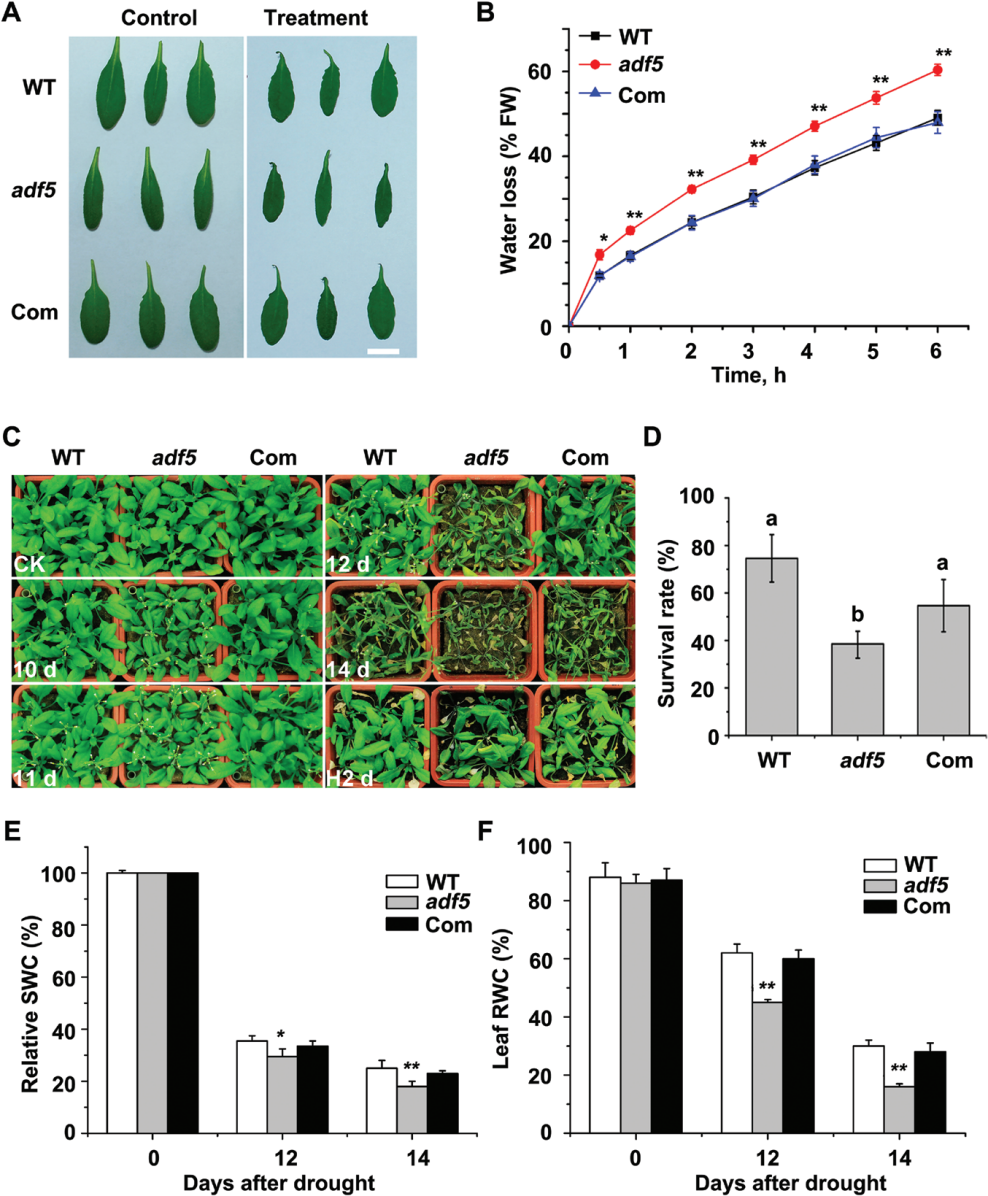
Mutation of *ADF5* increases plants’ sensitivity to water deficit. (A) Leaves detached from WT, *adf5*, and Com Arabidopsis plants were placed on a bench for 0 h (control) and 6 h (treatment). Scale bar=1 cm. (B) Water loss of leaves detached from WT, *adf5*, and Com plants. Data presented are the mean ±SE of three independent experiments. *P<0.05, **P<0.01 (Student’s *t*-test). (C) Phenotypic comparison of WT, *adf5*, and Com plants grown in soil after water was withheld for different durations (10, 11, 12, and 14 days) and the plants were then rewatered for 2 days. Three independent experiments were performed that yielded similar results. (D) Plant survival rate after rewatering for 3 days. Different letters above the bars represent significant (*P*<0.05) differences (Student’s *t*-test). Data presented are the means ±SEs of three independent experiments. (E) Relative soil water content (SWC) after withholding water for different durations. Data presented are the mean ±SD of three independent biological replicates. *P<0.05, **P<0.01 (Student’s *t*-test). (F) Leaf relative water content (RWC) after withholding water for different durations. Data presented are the mean ±SD of three independent biological replicates. **P<0.01 (Student’s *t*- test).

To determine whether ADF5 is actually involved in the response to abiotic stress, *adf5* T-DNA insertion mutants and *adf5* complementation (Com) lines were used to perform stress-related assays, as in our previous study (Zhu *et al.*, 2017). Compared with the WT plants, the *adf5* mutants had a higher rate of water loss during water stress (Fig. 2A, B). This experiment also revealed that the phenotype of the Com line was similar to that of the WT plants (Fig. 2A, B), suggesting that this phenotype is indeed caused by the loss of *ADF5*.

Next, the drought stress sensitivity of plants grown in soil was assessed to confirm the water loss *in vivo*. The WT and Com plants exhibited notable wilting after 12 days without watering, whereas the *adf5* mutant plants displayed increased wilting after 10 days without watering (Fig. 2C). All the plants were exposed to drought conditions for 15 days and then rewatered for 3 days, after which their survival rate was analyzed on the basis of the criteria that surviving plants could produce new leaves and grow normally. Compared with the WT plants, the *adf5* mutant plants had a lower survival rate, but the survival rate of the Com plants was similar to that of the WT plants (Fig. 2D). Consistent with these findings, relative SWC, which is related to water loss via transpiration in plants, and leaf RWC, which is an indicator of plant wilting caused by drought stress, were lower in the *adf5* plants than in the WT or Com plants after drought treatment (Fig. 2E). These results indicate that ADF5 plays an important role in the regulation of water loss during the plant response to drought stress.

### ABA-regulated stomatal movement is impaired by the *adf5* mutation

**Fig. 3.**
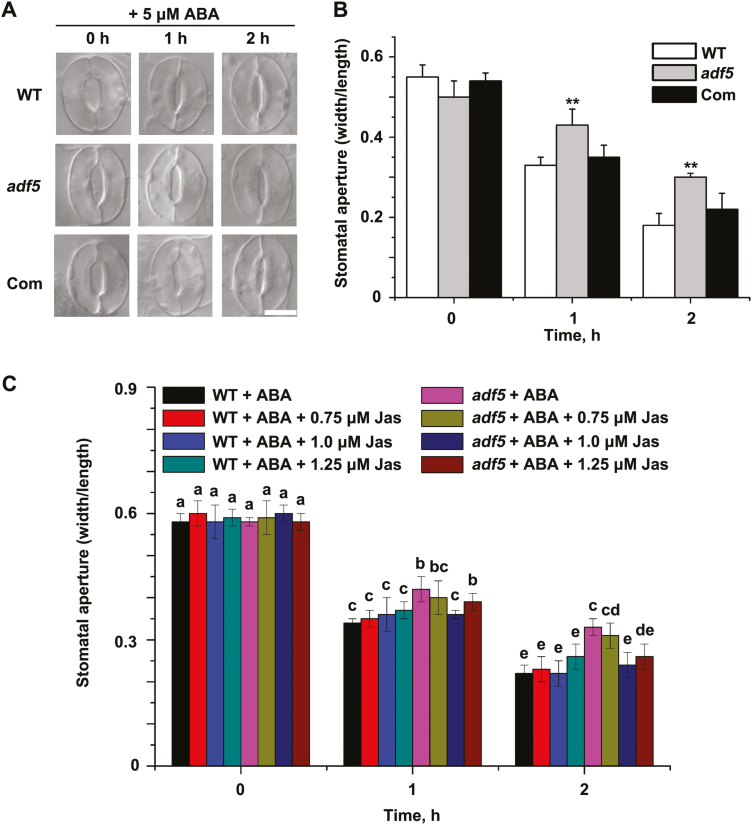
The *adf5* mutation impairs ABA-mediated stomatal closure. (A) Representative images of WT, *adf5*, and Com stomata showing stomatal closure in response to ABA treatment. (B) Quantification of stomatal closure in WT, *adf5*, and Com plants in response to 5 μM exogenous ABA; the stomatal aperture is indicated as the ratio of stomatal width/length. Data presented are the mean ±SE of three independent biological replicates. Significant differences in comparison with the WT are indicated as *P<0.05, **P<0.01 (Student’s *t*-test). (C) Jasplakinolide (Jas) partially rescued the stomatal sensitivity of *adf5* plants to ABA. The stomatal apertures were measured at the indicated times. Data presented are the mean ±SE of three independent biological replicates; different letters above the bars indicate significant (*P*<0.05) differences.

To determine the expression pattern of *ADF5*, we performed β-glucuronidase (GUS) staining on *ADF5*pro:*GUS* transgenic plants. As shown in Supplementary Fig. S2, GUS signals were detected in the leaves and stomatal guard cells, which was in agreement with the microarray data from AtGenExpress (Supplementary Fig. S3). To further investigate whether the drought sensitivity of the *adf5* plants resulted from impaired stomatal movement, we analyzed the ABA-induced stomatal closure. Following treatment with 5 µM ABA, the stomatal aperture of *adf5* plants was greater than in WT plants (Fig. 3A, B), suggesting that stomatal closure in response to ABA was impaired in the *adf5* plants. The phenotype of the Com plants was similar to that of the WT plants during ABA-induced stomatal closure (Fig. 3A, B), suggesting that this phenotype is indeed caused by the loss of function of *ADF5*. Thus, the disruption of *ADF5* expression impaired stomatal closure in response to ABA. Interestingly, the physiological function of ADF5 is the opposite of that of ADF4 during stomatal movement (Zhao *et al.*, 2016).

Actin reorganization is essential for stomatal closure (Zhao *et al.*, 2011, 2016; Jiang *et al.*, 2012). Given that ADF5 can bind and bundle actin filaments (Nan *et al.*, 2017), we proposed that the dynamic changes in actin regulated by ADF5 were essential for ABA-induced stomatal closure. Thus, actin-related pharmacological assays were performed. Initially, we explored the effects of a series of concentrations of jasplakinolide (Jas), an actin stabilizer that can bind and stabilize actin filaments (Fig. 3C), on stomatal aperture The stomatal sensitivity of *adf5* plants to ABA recovered to that of the WT plants after Jas treatment. These results indicate that the F-actin-bundling activity of ADF5 might play a vital role in altering stomatal aperture in response to ABA.

### The *adf5* mutation delays actin reorganization during stomatal closure

**Fig. 4.**
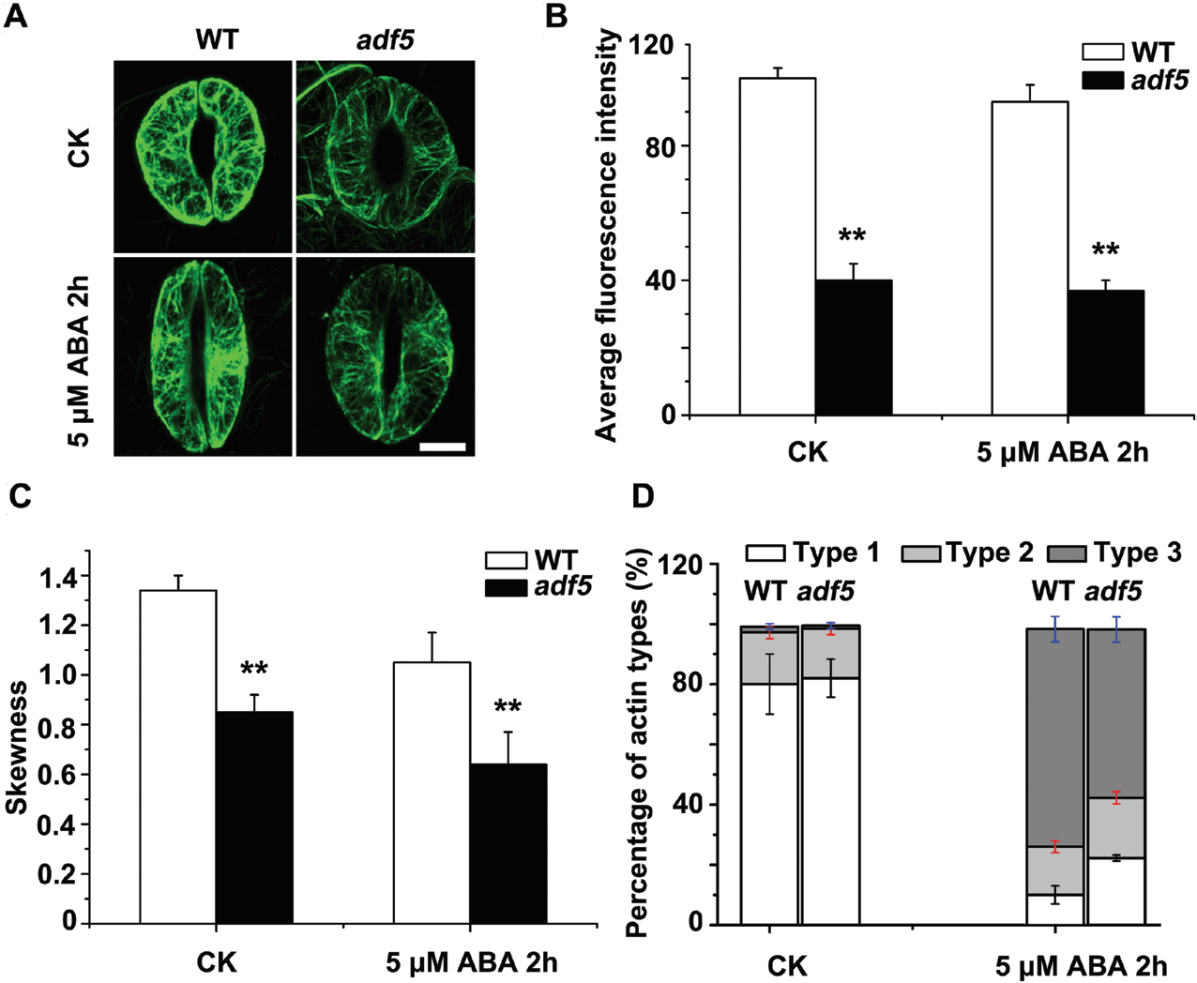
The loss of function of *ADF5* delays actin reorganization during stomatal closure. (A) Confocal images of guard cells in rosette leaves from FABD2 (WT) and *adf5*×FABD2 transgenic plants after treatment with 5 μM ABA at 0 h (control, CK) and 2 h, showing GFP-labeled actin filaments. Scale bar=5 μm. (B) Quantification of the intensity of fluorescence of the GFP signal of WT and *adf5* guard cells. (C) Quantification of bundling (skewness) of actin filaments in WT and *adf5* guard cells. (D) Analysis of the type of actin organization in guard cells: type 1, radial array; type 2, random meshwork; type 3, longitudinal array. Data presented are the mean ±SE of three independent biological replicates. At least 60 stomata were analyzed for each time point. *P<0.05, **P<0.01 (Student’s *t*-test).

To further investigate how impaired stomatal closure in the *adf5* mutant was related to altered actin dynamic organization and arrangement *in vivo*, we examined the actin structures in guard cells derived from WT plants and *adf5* plants expressing FABD2-eGFP. The actin filaments appeared as thick, radially oriented bundles in the guard cells of the WT plants in the open state, consistent with the results of a previous study (Jiang *et al.*, 2012), whereas in the *adf5* plants under the same conditions the actin filaments appeared as thinner bundles in the guard cells (Fig. 4A). To quantify the actin filaments and the extent of actin bundling, we first quantified the intensity of fluorescence of actin filaments in WT and *adf5* guard cells following treatment with ABA. We observed that, relative to the WT plants, the fluorescence intensity of the guard cells of *adf5* plants was significantly lower after ABA treatment (Fig. 4B). We then measured the skewness of the actin filaments and observed that it was significantly lower in *adf5* plants than in the WT plants (Fig. 4C).

To quantitatively analyze the rearrangement of actin in guard cells in response to ABA, we classified the actin organization into three distinct patterns, termed type 1, type 2, and type 3 (Zhao *et al.*, 2011; Jiang *et al.*, 2012). Before ABA treatment, the WT and *adf5* guard cells exhibited similar patterns of actin organization, and most patterns were type 1, with sparse, transversely oriented actin filaments (Fig. 4D). After 2 h of ABA treatment, the proportion of type 3 actin increased dramatically in the guard cells of both WT (from 2.1% to 72.1%) and *adf5* (from 0.9% to 58.9%) plants (Fig. 4D); the proportion of type 1 actin in the WT guard cells decreased to 10.3%, while in the *adf5* guard cells the decrease was less pronounced, to 22.1% (Fig. 4D). Taken together, these results suggest that the *adf5* mutation delays the reorganization of actin filaments during stomatal closure.

### Up-regulation of *ADF5* expression by ABA depends partly on DPBF3

**Fig. 5.**
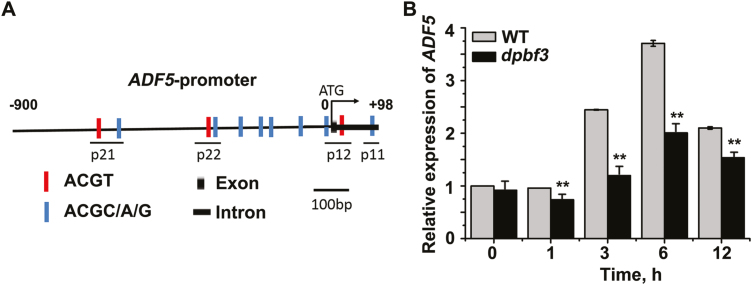
Up-regulation of *ADF5* expression by ABA partly depends on DPBF3. (A) Schematic diagram of the *ADF5* promoter. (B) Expression levels of *ADF5* by qRT–PCR analysis in WT and *dpbf3* plants subjected to ABA treatment. Twelve-day-old seedlings were treated with 40 μM ABA for different durations, after which the leaves of the seedlings were detached for RNA extraction. *UBQ11* was used as an internal standard. Data presented are the mean ±SD of three independent biological replicates. *P<0.05, **P<0.01 (Student’s *t*-test).

To study the potential upstream factor(s) of ADF5, we first analyzed the *cis*-acting elements of the *ADF5* promoter related to ABA signaling. As shown in Fig. 5A and Supplementary Fig. S4, the *ADF5* promoter contains several ACGT/C sequences, the core motif of ABRE, indicating that ABA may regulate the expression of *ADF5* by ABF/AREB transcription factors via the G-box, as members of this transcription factor family are involved predominantly in the ABA signaling response (Guiltinan *et al.*, 1990; Busk and Pagès 1998; Kim *et al.*, 2016). Among the nine members of this family, only *ABF1*, *ABF2*, *ABF3*, *ABF4*, *DPBF3*, and *DPBF4* are expressed mainly in leaves.

In addition, the transcription levels of *ADF5* were evaluated in WT plants and in *abf1*, *abf2*, *abf3*, *abf4*, *dpbf3*, *and dpbf4* mutant plants by qRT–PCR analysis. The results showed that the transcription levels of *ADF5* were significantly lower than WT in *abf1*, *abf2*, *abf3*, *abf4*, and *dpbf3* mutant plants, but not in the *dpbf4* mutant (Fig. 5B; Supplementary Fig. S5). These results indicate that ABA-induced expression of *ADF5* depends partly on ABF/AREBs.

### DPBF3 binds directly to the *ADF5* promoter and activates its transcription

A previous study (Yoshida *et al.*, 2015) revealed no significant differences in stomatal aperture between *abf1*/*abf2*/*abf3*/*abf4* quadruple mutants and WT plants, suggesting that ABF1, ABF2, ABF3, and ABF4 may not participate in stomatal movement. On the basis of the above data, we examined the potential role of DPBF3 in the regulation of *ADF5* expression in response to ABA.

**Fig. 6.**
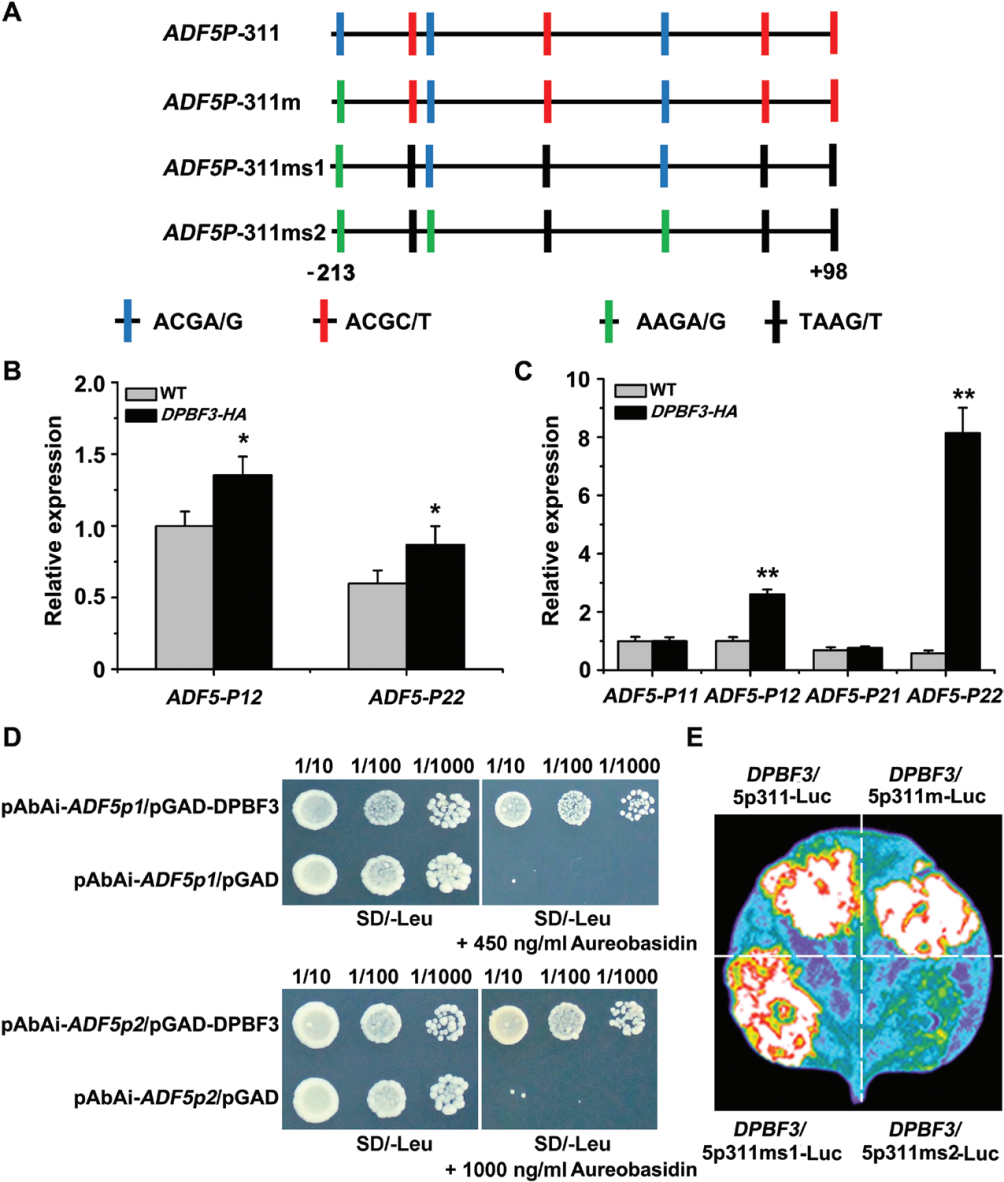
DPBF3 binds directly to the *ADF5* promoter and activates *ADF5* expression. (A) Schematic diagram of the motifs in the *ADF5* promoter. ADF5p311 indicates the 311 bp region downstream of the *ADF5* promoter, and ADF5p311m indicates the change from ACGA/G to AAGA/G at position –203 to –206 bp. Similarly, ADF5p311ms1 indicates the change from ACGC/T to TAAG/T at positions –5 to –78 bp, –147 to –150 bp, +44 to +47 bp, and +94 to +97 bp; and ADF5p311ms2 indicates the change from ACGC/T to TAAG/T at positions –75 to –78 bp, –147 to –150 bp, +44 to +47 bp, and +94 to +97 bp, and the change from ACGA/G to AAGA/G at positions –1 to –4 bp, –135 to –138 bp, and –203 to –206 bp. (B) ChIP analysis of the interaction between DPBF3 and the *ADF5* promoter under normal conditions. Fragment P12 localizes to –10 to +55 bp and P22 localizes to –200 to –270 bp of the *ADF5* promoter. (C) ChIP analysis of the interaction between DPBF3 and the *ADF5* promoter after treatment with 40 μM ABA. Fragment P11 localizes to +75 to +98 bp and P21 localizes to –500 to –635 bp of the *ADF5* promoter. Data presented are the mean ±SD of three independent biological replicates. *P<0.05, **P<0.01 (Student’s *t*-test). (D) Y1H assay of the interaction between DPBF3 and the *ADF5* promoter, showing the growth of yeast cells on 450 and 1000 ng ml^–1^ aureobasidin A-SD/Leu medium. Cells were grown in liquid medium to an OD_600_ of 1.0. The numbers above the images indicate the dilutions. (E) DPBF3 could activate the expression of *ADF5* when transiently expressed in tobacco leaves.

ChIP experiments were performed to determine whether DPBF3 bound to the *ADF5* promoter *in vivo*. Chromatin immunoprecipitated with the anti-HA antibody was enriched in fragments P12 (–10 to +55 bp) and P22 (–200 to –270 bp) of the *ADF5* promoter in *35S*:*DPBF3*-*HA* seedlings (Fig. 6A, B; Supplementary Fig. S6), and the interaction of DPBF3 with the *ADF5* promoter was enhanced after ABA treatment (Fig. 6C). Furthermore, there was almost no enrichment in the WT plants or in fragments P11 (+75 to +98 bp) and P21 (–500 to –635 bp) of the *ADF5* promoter in *35S*:*DPBF3*-*HA* seedlings (Fig. 6B, C), indicating that DPBF3 bound to the promoter of *ADF5 in vivo*. Y1H assays were then performed, which confirmed that DPBF3 could bind directly to the *ADF5* promoter in the yeast *in vitro* (Fig. 6D). These results confirmed that *ADF5* is a target gene of DPBF3.

To further assess the function of DPBF3 in the regulation of *ADF5* expression, we performed a transient expression experiment in tobacco leaves. DPBF3 could activate the expression of *ADF5*, and only mutation of all the ACG motifs abolished the transcriptional activation by DPBF3 (Fig. 6A, E), suggesting that DPBF3 activates the expression of *ADF5* via the ACG motifs. These data indicate that DPBF3 can directly activate and regulate the expression of *ADF5* following ABA induction.

### DPBF3 may be redundant with other ABF/AREBs in the regulation of stomatal movement

**Fig. 7.**
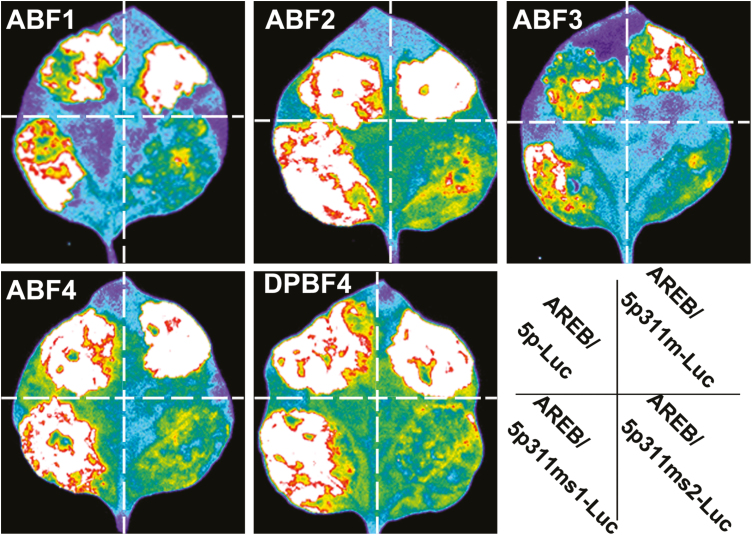
ABF1, ABF2, ABF3, ABF4, and DPBF4 can all activate the expression of *ADF5* via the ABRE core motif when transiently expressed in tobacco leaves. Leaves of *N. benthamiana* were co-infiltrated with ADF5p311-Luc, the 311 bp region downstream of the *ADF5* promoter tagged with firefly luciferase. ADF5p311m indicates the change from ACGA/G to AAGA/G at position –203 to –206 bp. Similarly, ADF5p311ms1 indicates the change from ACGC/T to TAAG/T at positions –5 to –78 bp, –147 to –150 bp, +44 to +47 bp, and +94 to +97 bp; and ADF5p311ms2 indicates the change from ACGC/T to TAAG/T at positions –75 to –78 bp, –147 to –150 bp, +44 to +47 bp, and +94 to +97 bp, and the change from ACGA/G to AAGA/G at positions –1 to –4 bp, –135 to –138 bp, and –203 to –206 bp. All mutations in ACG motifs abolished the binding between AREB/ABF transcription factors and the promoter of *ADF5*.

To determine whether DPBF3 is involved in stomatal movement, WT plants and *dpbf3* mutants were used to analyze stomatal closure in response to ABA treatment. The stomatal closure in *dpbf3* plants was similar to that in WT plants under ABA treatment (Supplementary Fig. S7), indicating that DPBF3 may be redundant with other ABF/AREBs. Therefore, we crossed *dpbf3* with *dpbf4* to obtain a *dpbf3*/*4* double mutant (Supplementary Fig. S8), and found that there were no differences in stomatal closure between the WT line and the *dpbf3*/*4* double mutant (Supplementary Fig. S7). Furthermore, using transcriptional activation experiments, we also tested whether ABF1, ABF2, ABF3, ABF4, and DPBF4 could activate the expression of *ADF5* via the conserved ABRE core site. ABF1, ABF2, ABF3, ABF4, and DPBF4 all could activate the expression of *ADF5* via the ACG motifs (Fig. 7). Taken together, these results indicate that ABF/AREBs might be redundant among each other in the regulation of stomatal movement.

## Discussion

### Neofunctionalized ADF5 improves Arabidopsis resistance to drought stress via its actin-stabilizing activity in the regulation of stomatal closure

Phylogenetic analyses have revealed that the ADF variants are clustered into four ancient subclasses in plant lineages (Nan *et al.*, 2017). In *A. thaliana*, biochemical analyses have revealed that all 11 ADF proteins exhibit opposing biochemical properties: subclass I/II/IV ADFs display conserved F-actin severing/depolymerizing (D-type) activities, while subclass III ADFs evolved F-actin-bundling (B-type) function (Zheng *et al.*, 2013; Nan *et al.*, 2017; Zhu *et al.*, 2017). However, the importance of the physiological function and evolution of neofunctionalized ADFs is not well elucidated. In this study, we found that neofunctionalized ADF5 is involved in the ABA/drought signaling pathway. In addition, the loss of function of *ADF5* inhibited stomatal closure by decreasing F-actin bundling in guard cells (Figs 3 and 4), suggesting that ADF5 promotes stomatal closure via F-actin bundling in guard cells. Our pharmacological assays also revealed that Jas could partially restore the stomatal sensitivity of *adf5* plants to ABA (Fig. 3C); however, we cannot rule out the possibility that such processes may occur as indirect effects of altering the G/F-actin pool.

On the other hand, the conserved subclass I ADF4 displays F-actin severing/depolymerizing activities, and the loss of function of *ADF4* promotes stomatal closure in response to drought stress (Zhao *et al.*, 2016), indicating that ADF4 inhibits stomatal closure by severing/depolymerizing F-actin in plants. Tholl *et al.* (2011) reported that typical ADF1 and neofunctionalized ADF9 regulate or modulate actin dynamics in an opposing manner and compete with each other, implying that plants evolved neofunctionalized ADFs to regulate actin dynamics synergistically and tightly. Therefore, we speculated that neofunctionalized ADF5, in coordination with conserved ADF4, evolved to help Arabidopsis resist drought stress by regulating stomatal movement.

### Transcriptional regulation may be a complementary ABA signaling pathway for the activity of ADFs during stomatal movement

Pharmacological and genetic studies have demonstrated that remodeling of the actin cytoskeleton is essential for ABA/drought-induced stomatal closure; however, the potential mechanism linking them remains poorly understood. Generally, stomatal movement is a fast process and takes less than 30 min, while gene transcription to translation takes 1–2 h. Previous studies concerning ADFs have focused mostly on their post-translational regulation during stomatal movement. For example, Zhao *et al.* (2016) reported that ADF4 mediates ABA signaling and actin cytoskeleton remodeling via phosphorylation during stomatal closure, and the Ser-6 phosphorylation site is conserved in several plant ADFs (Allwood *et al.*, 2002; Chen *et al.*, 2002; Zhao *et al.*, 2016), indicating that phosphorylation may be an important means of tailoring ADF activity in regulating stomatal closure in response to drought stress.

**Fig. 8.**
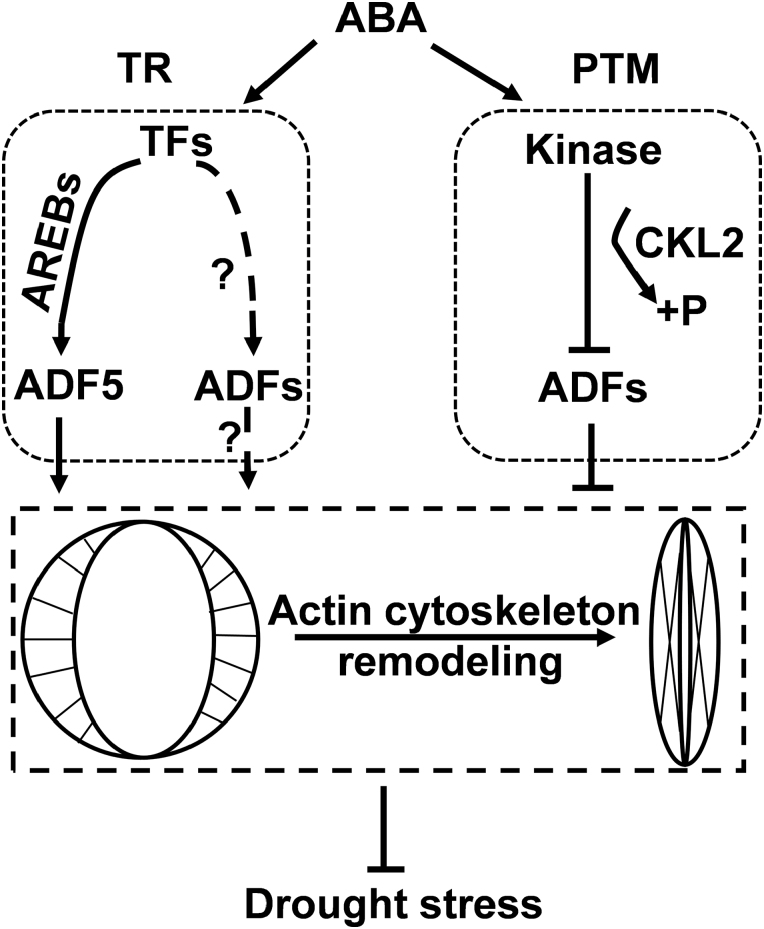
A working model for ADFs involved in the regulatory network between ABA signaling and actin cytoskeleton remodeling. ABA/drought activates downstream kinases such as CKL2, which phosphorylate ADFs (ADF1/2/3/4) and repress their actin severing/depolymerizing activity. This cascade disrupts actin cytoskeleton dynamics and subsequently inhibits stomatal closure via post-translational modification (PTM). Transcriptional regulation (TR) may be complementary to the ABA signaling pathway for the activity of ADFs during stomatal movement. Transcription factors (TFs) such as AREB/ABFs up-regulate the expression of *ADF5*, which stabilizes actin filaments to promote stomatal closure. Additional ADFs (ADF1/2/3/4/6) may also be involved in ABA-regulated stomatal closure via TR.

In this study, we observed that DPBF3, a member of the ABF/AREBs subclass, binds to the *ADF5* promoter and further activates *ADF5* transcription via the ABRE core motif ACGT/C (Fig. 6). These results revealed that ABA signaling regulates *ADF5* expression at the transcriptional level, after which ADF5 remodels the actin cytoskeleton during stomatal movement. ABF/AREB transcription factors and ADF5 may represent a potential link between ABA signaling and the actin cytoskeleton during stomatal closure. Genome sequencing analysis revealed that all promoters of *ADFs* expressed in vegetative tissues of Arabidopsis, with the exception of the promoter of *ADF9*, contain the ABRE core motif ACGT/C (Supplementary Fig. S9), which is the core binding site of ABF/AREB transcription factors. qRT–PCR analyses revealed that expression of these *ADF*s was also induced by ABA (Supplementary Fig. S10), indicating that Arabidopsis ADFs jointly participated in the ABA signaling pathway via transcriptional regulation. Thus, the transcriptional regulation of *ADF*s might be a complementary pathway for ABA-regulated stomatal closure. Therefore, ADFs may regulate stomatal movement in response to ABA and drought stress via at least two pathways: the expression level of *ADF*s by transcriptional regulation, and the activity of ADFs by phosphorylation (Fig. 8).

Compared with *ADF* expression in the WT plants, *ADF5* expression in the *abf*/*areb* single mutant in response to ABA treatment was significantly lower (Fig. 5B; Supplementary Fig. S5), and ABF1, ABF2, ABF3, ABF4, and DPBF4 could activate the expression of *ADF5*. However, recent studies have suggested that the water loss rate of Arabidopsis *abf1*/*abf2*/*abf3*/*abf4* quadruple mutants was only slightly (and not significantly) increased compared with that of Arabidopsis WT plants (Yoshida *et al.*, 2010, 2015). Additionally, DPBF3 partially repressed *ADF5* expression, and the water loss of *dpbf3*/*4* double mutant plants did not markedly differ from that of WT plants (Fig. 5B; Supplementary Fig. S7). These results indicate that relatively low *ADF5* expression may be sufficient to maintain its function in regulating stomatal closure. Additionally, we cannot rule out the possibility that the function of ABF/AREBs during stomatal closure is redundant with that of other family members. Therefore, the expression of *ADF5* in response to ABA signaling may be completely abolished in *abf*/*arebs* sextuple or more mutant plants. To summarize, ADFs may be new transcriptional-level components of ABA signaling in guard cells.

## Supplementary data

Supplementary data are available at *JXB* online.

**Table S1.** Information on the primers used in this study.

**Fig. S1.** *ADF5* expression is induced by ABA according to the results of a microarray analysis.

**Fig. S2.** Analysis of *ADF5* expression patterns.

**Fig. S3.** *ADF5* expression in leaves and guard cells.

**Fig. S4.** Predicted ABRE core motif and GCEs in the *ADF5* promoter.

**Fig. S5.** Expression of *ADF5* in WT plants and *abf*/*areb* mutant plants.

**Fig. S6.** Identification of *DPBF3* overexpression transgenic lines.

**Fig. S7.** Phenotypic comparison of stomatal closure and water loss among WT, *dpbf3*, *dpbf4*, and *dpbf3*/*4* plants.

**Fig. S8.** Identification of the T-DNA insertion in *dpbf3*/*4* homozygous mutants.

**Fig. S9.** Prediction of the ABRE core motif and GCEs in the *ADF*s promoter.

**Fig. S10.** Expression levels of *ADFs* induced by ABA.

## Data deposition

Sequence data from this article can be found in the Arabidopsis Information Resource (https://www.arabidopsis.org/) or GenBank (http://www.ncbi.nlm.nih.gov/genbank/) databases under the following accession numbers: ADF1/AT3G46010, ADF2/AT3G46000, ADF3/AT5G59880, ADF4/AT5G59890, ADF5/AT2G16700, ADF6/AT2G31200, DPBF1/ABI5/AtbZIP39/At2g36270, DPBF2/AtbZIP67/At3g44460, DPBF3/AREB3/AtbZIP66/At3g56850, DPBF4/EEL/AtbZIP12/At2g41070, DPBF5/ABF3/AtbZIP37/At4g3400, AREB1/ABF2/AtbZIP36/At1g45249, AREB2/ABF4/AtbZIP38/At3g19290, ABF1/AtbZIP35/At1g49720, AtbZIP15/At5g42910.

## Supplementary Material

Supplementary Fig. S1

Supplementary Fig. S2

## Abbreviations:

ABA: abscisic acid
ABP: actin-binding protein
ABRE: ABA-responsive element
ADF: actin-depolymerizing factor
bZIP: basic leucine zipper
ChIP: Chromatin immunoprecipitation assay
F-actin: actin filaments
RWC: relative water content
SWC: soil water content
Y1H: yeast one-hybrid.
