# Supplementary material for: Arabidopsis ADF5 promotes stomatal closure by regulating actin cytoskeleton remodeling in response to ABA and drought stress
Source: J Exp Bot. 2018 Nov 24;70(2):435–46. doi: 10.1093/jxb/ery385 (PMC6322581; doi:10.1093/jxb/ery385)
Supplement: Supplementary Fig. S1 [file ery385_suppl_supplementary-fig-s1.pdf]

Table S1. Primer information used in this study

Primer name	Sequence (5'-3')
DPBF3-F	GGGGACAAGTTTGTACAAAAAAGCAGGCTTCATGG ATTCTCAGAGGGGTATTG
DPBF3-R	GGGGACCACTTTGTACAAGAAAGCTGGGTCGAAAG GAGCCGAGCTTGTCCGT
ADF5p1-F	AAGCTTATGACTTTAAGTGCTTTAT
ADF5p1-R	GGTACCTCTATATTTTTTTTTTCAA
ADF5p2-F	AAGCTTTTTGATTTAAATTTATTT
ADF5p2-R	GGTACCTACAAAGTGAGCAACTTCAC
ADF5P-331-F	GGGGACAAGTTTGTACAAAAAAGCAGGCTTCTGCCG ACGAGAAAAAGAAC
ADF5P-331-R	GGGGACCACTTTGTACAAGAAAGCTGGGTCCTGCGAA TCATCATTAGCTA
DPBF3-Y-F	GGATCCATGGATTCTCAGAGGGGTAT
DPBF3-Y-R	CTCGAGGAAAGGAGCCGAGCTTGTC
ADF5-F	GGGGACAAGTTTGTACAAAAAAGCAGGCTTCATGGC GATGGCTTTCAAG
ADF5-R	GGGGACCACTTTGTACAAGAAAGCTGGGTCTTTGGCA CGGTCTTGGAT
ABF1-F	GGGGACAAGTTTGTACAAAAAAGCAGGCTTCATGGG TACTCACATTGATATC
ABF1-R	GGGGACCACTTTGTACAAGAAAGCTGGGTCCCTTCTT ACCACGGACCGGTA
ABF2-F	GGGGACAAGTTTGTACAAAAAAGCAGGCTTCATGGA TGGTAGTATGAATTTG
ABF2-R	GGGGACCACTTTGTACAAGAAAGCTGGGTCCCAAGG TCCCGACTCTGTCCT
ABF3-F	GGGGACAAGTTTGTACAAAAAAGCAGGCTTCATGG ATTCTCAGAGGGGTATTG
ABF3-R	AAGAAAGCTGGGTCCCAGGGACCCGTCAATGTCCTT
ABF4-F	GGGGACAAGTTTGTACAAAAAAGCAGGCTTCATGGG AACTCACATCAATTTC
ABF4-R	GGGGACCACTTTGTACAAGAAAGCTGGGTCCCATGG TCCGGTTAATGTCCTT

DPBF4-F	GGGGACAAGTTTGTACAAAAAAGCAGGCTTCATGG GTTCTATTAGAGGAAAC
DPBF4-R	GGGGACCACTTTGTACAAGAAAGCTGGGTCGAGAG AAGCAGAGTTTGTTC
ADF5-Chip-11-F	TTAAAGATTCTTGATCTGAT
ADF5-Chip-11-R	CTGCGAATCATCATTAGCTAT
ADF5-Chip-12-F	CTTTGTAACCCTTTAGCAGCAA
ADF5-Chip-12-R	CTTTAAACGTAGAAAAACAAAT
ADF5-Chip-21-F	ATGCCGACGAGAAAAAGC
ADF5-Chip-21-R	AAATGCGATCACTATTTACTC
ADF5-Chip-22-F	GCAAATAAATCGTTAGATATT
ADF5-Chip-22-R	ATTAACGCACCGTGTGTTCTG
adf5-LP	AAATGCTTAAACACGACACGG
adf5-RP	GCTACGCTGTCTTTGATTTCG
dpbf3-LP	TCTTCTTTGATGGGTGGTTTG
dpbf3-RP	CAAGCTTGGCTATTGCAGAAC
dpbf4-LP	TCCACTTAGGATCTGGTGGTG
dpbf4-RP	ATACTGCATGCAAATTCCCTG
LBb1.3	ATTTTGCCGATTTCGGAAC
ADF5-qRT-F	CGTTTGTGTTTTGATTGTGTTGTTAA
ADF5-qRT-R	CCGTTACTCGTAGGACAAATTCG
DPBF3-qRT-F	GAGAGTCTGCTGCTCGTTCCCG
DPBF3-qRT-R	TTCCACCTCCTTTTGCTTCCTG
DPBF4-qRT-F	ATAATCCACCCATCATCACCAT
DPBF4-qRT-R	TCATCAGCCTCAACAACAACAA
UBQ11-qRT-F	CACACTCCACTTGGTCTTGCGT
UBQ11-qRT-R	TGGTCTTTCCGGTGAGACTCTTCA
RD29A-qRT-F	GTTACTGATCCCACCAAAGAAGA
RD29A-qRT-R	GGAGACTCATCAGTCACTTCCA
