# Supplementary material for: Arabidopsis ADF5 promotes stomatal closure by regulating actin cytoskeleton remodeling in response to ABA and drought stress
Source: J Exp Bot. 2018 Nov 24;70(2):435–46. doi: 10.1093/jxb/ery385 (PMC6322581; doi:10.1093/jxb/ery385)
Supplement: Supplementary Fig. S2 [file ery385_suppl_supplementary-fig-s2.pdf]

Fig. S1

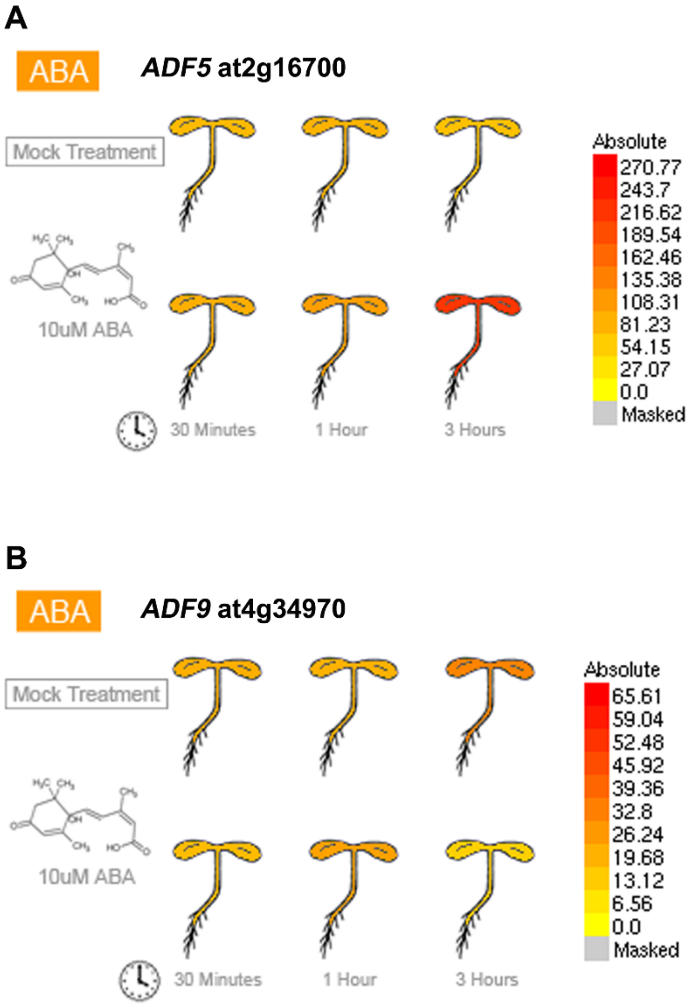

Fig. S1. *ADF5* expression is induced by ABA from microarray analysis.

(A) The expression level of *ADF5* is upregulated by ABA from the e-FP browser (http://bbc.botany.utoronto.ca/efp/cgi-bin/efp). Red indicates high expression, and blue scale denotes low expression. (B) The expression levels of ADF9 after ABA treatment from the e-FP browser (http://bbc.botany.utoronto.ca/efp/cgi-bin/efp). Red indicates high expression, and blue scale denotes low expression.

Fig. S2

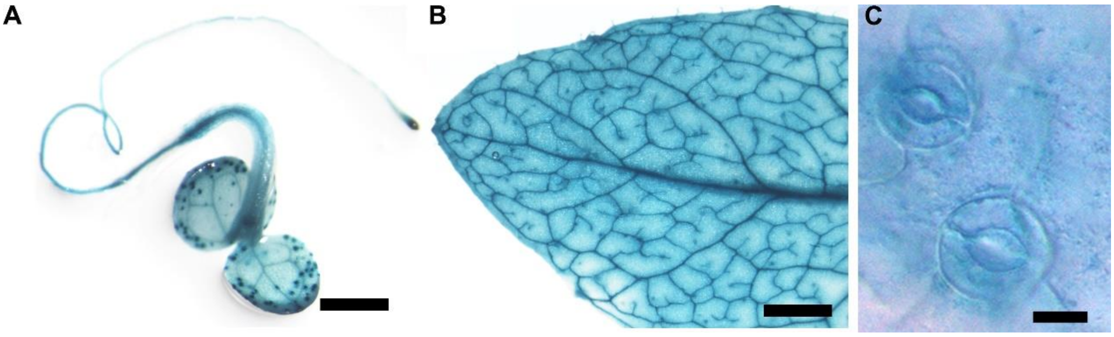

Fig. S2. Analysis of ADF5 expression patterns.

The GUS activity in the PBI121-ADF5Pro-GUS transgenic plants in cotyledon (A), rosette leaf (B) and guard cells (C).

Fig. S3

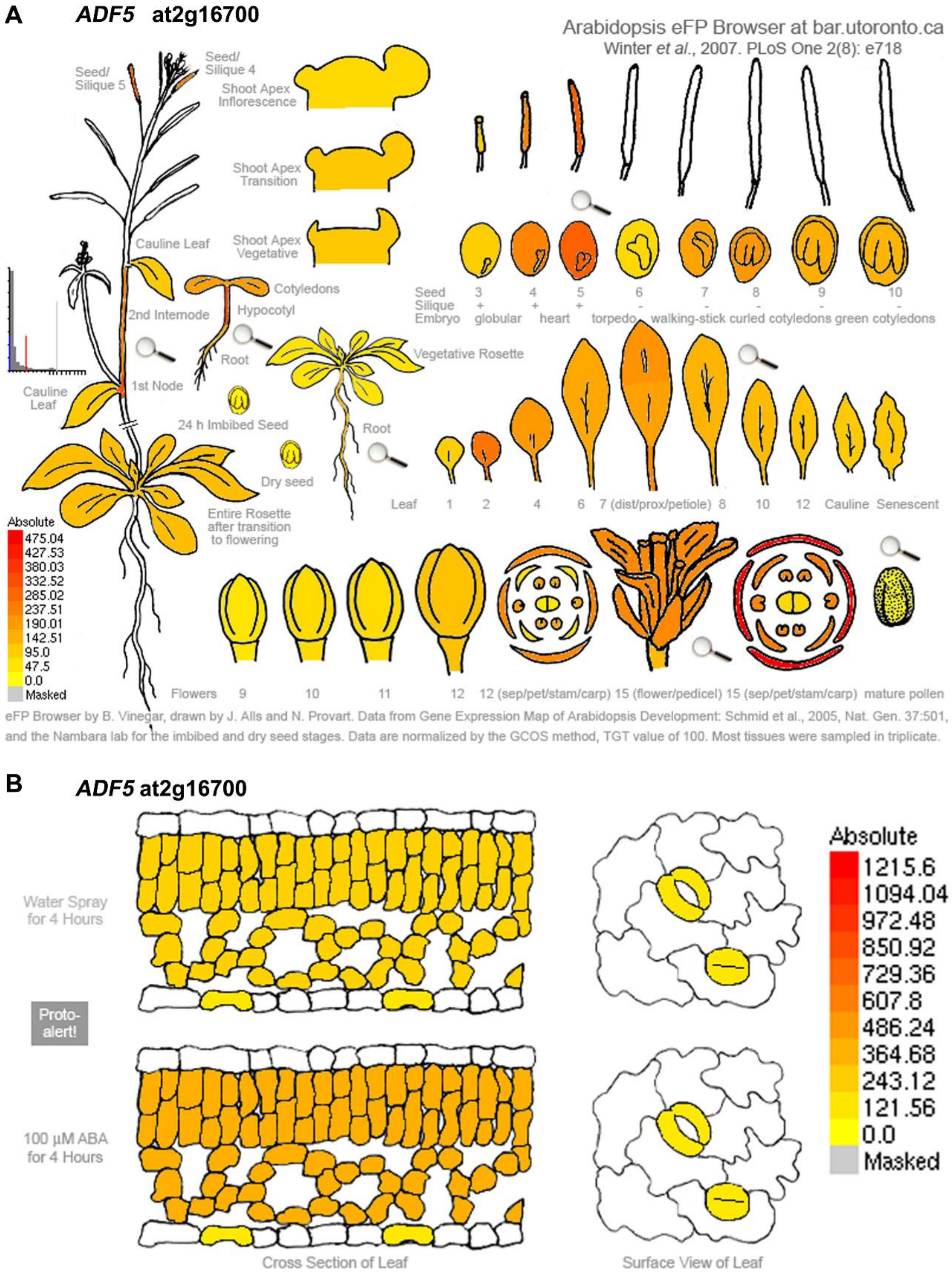

Fig. S3. *ADF5* expression in leaves and guard cells.

(A) and (B) The expression level of *ADF5* in leaves and guard cells from the e-FP browser (http://bbc.botany.utoronto.ca/efp/cgi-bin/efp). Red indicates high expression, and blue denotes low expression.

Fig. S4

aaaatatatataaaaaaaattattagatatttatcagaaaaagacaagatttattagtgcagaaca 66

cacggtgacgttaatatgccgacgagaaaagaacaaaaatattcgtctctaacaaaaaagttt 132

acaagggtgagtttgtaataagagtaaatagtgatcgcatttgtataggcataactttttatataaa 198

gacctcaaagtcttaccatttgaacagaaaaaagctctccacgcctaaaaaaaaagaaaaaa 264

gagacagaagaagaaaaagataaagaagaatcaaactttgtaaccctttagcagcaATGG 330

CGATGGCTTTCAAGATGgtaaattcaaatttgtttttctacgtttaaagattcttgatctgat 396

ctttaaacaaatagctaatgatgattcgcag 427

Fig. S4. The prediction of ABRE core motif and GCEs in the *ADF5* promoter.

The prediction of ABRE core motif and GCEs in the *ADF5* promoter.

Fig. 5

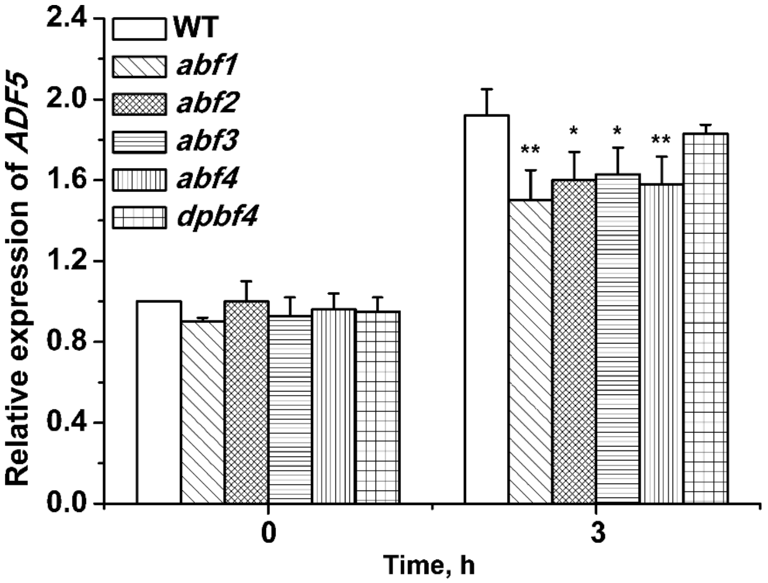

Fig. S5. Expression of *ADF5* in WT and *abf/areb* mutant plants.

qRT-PCR analysis revealed *ADF5* expression by ABA. Fourteen-day-old seedlings were treated with 40 μM ABA for different times, and the leaves of seedling were detached for RNA extraction. *UBQ11* was used as an internal gene.

Fig. S6

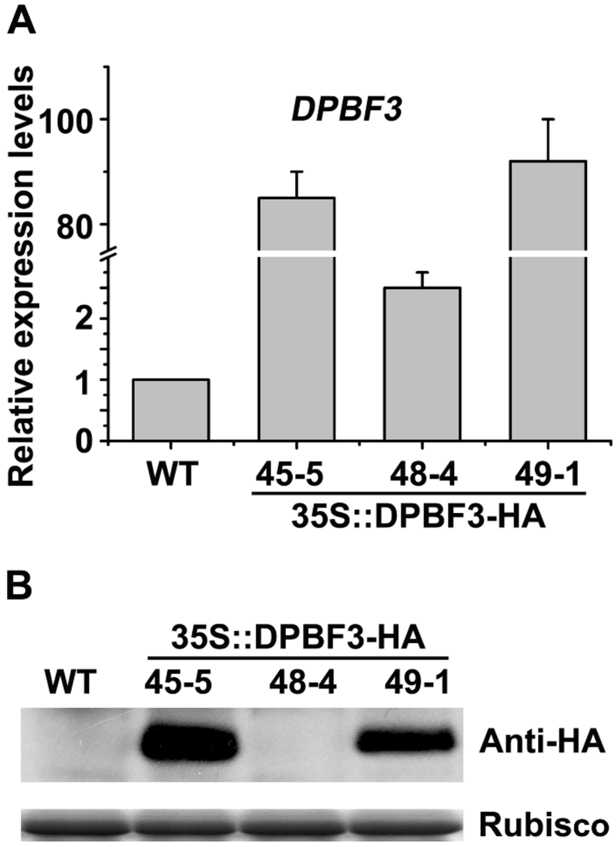

Fig. S6. Identification of the DPBF3 overexpression transgenic lines.

(A) qRT-PCR analysis of *DPBF3* expression in 35S:DPBF3-HA overexpression plants.

(B) Immunoblot analysis of DPBF3 by HA monoclonal antibody in WT and 35S:DPBF3-HA overexpression plants. Rubisco stained by Coomassie brilliant blue was used as an internal loading control.

Fig. S7

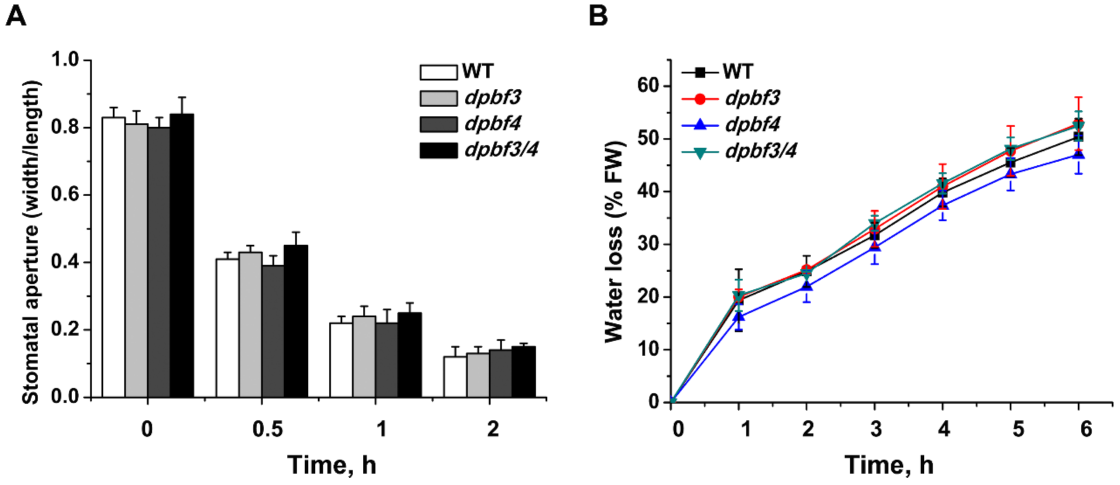

Fig. S7. Phenotypic comparison of stomatal closure and water loss among WT, *dpbf3*, *dpbf4* and *dpbf3/4* plants.

(A) The phenotype comparison of the stomatal closing rate of WT, *dpbf3*, *dpbf4* and *dpbf3/4* plants induced by ABA. (B) Analysis of water loss from the detached leaves of WT, *dpbf3*, *dpbf4* and *dpbf3/4* plants. The data represent the mean ± SE based on three independent biological replicates.

Fig. S8

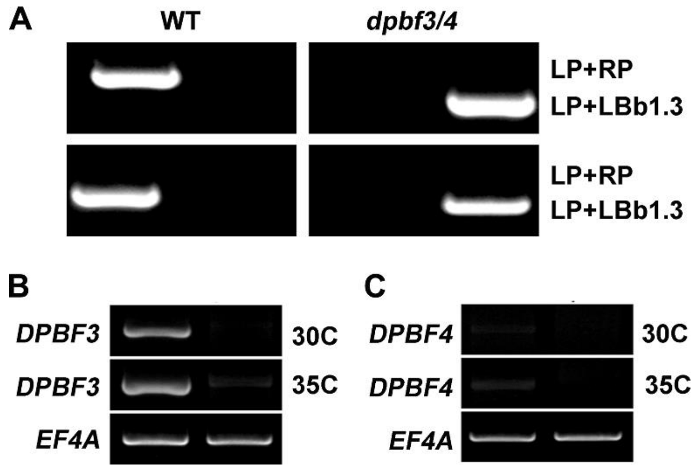

Fig. S8. Identification of the T-DNA insertion in the *dpbf3/4* homozygous mutants. (A) *dpbf3/4* homozygous T-DNA insertion mutant identification by PCR. LP and RP are mutant-specific gene primers, and LBb1.3 indicates the T-DNA left border primer. (B) and (C) RT-PCR analysis of *DPBF3* and *DPBF4* expression in WT and *dpbf3/4* double mutant lines.

Fig. S9

ADF1

tattttttgtagttgttgtggtgttacacatcaatattttttacttcttaaaagttgaatcaagttctcaaaccaacaaactttttatcga
atcaatggtacgacgaaattcaaaaatgttattacttactaattttatagatagtttaaaaatataaattaaaaattcagtttatttata
taacaagaaaaatcagtgttattattgaaacacctaccaaaataatgaatcaaaaacataaaattaattatagtattgaattcata
attcaacaatattattgaaaatttgaaatacataccaaataataatagttttttttcgtctctataatatggtggactcccgaataaa
gttgaagaaagaaagagaaaagaaattgagaagaaaaaagaaaaaaaggtgaccgtattctccgtcgattgtagccgccc
gaagaaatatcttctcctctctcgtttaacccacgaatttgctctgatattagtgtttctctagccATGGTAAGCTCAA
TTTTCCACTTGTTATCTCGATCTgtagtgtgcttctctcgattttggtttcgtcttatatacaaatcttctgttt
acgcgattctggctcattttgttgtttcgctagatctgtatacagattttggttttagatctctaagatttggtgtttatgtgatcgtttt
gtgactgatttagaatcaaaactgtgcgagagagcttctctgttgagttcactgtggctctttcgattcctctatctaaaatttgaa
atcagaagagtatttacggcttcctgttattgcaaaaaaatttcaaagttatatatcaatttcgataacatttggttgtttgatccatta
tccttgatgtttgattcatcagtttcatgtaattgataatcactctagaggtttgagaacaatagaggtttttggattgtgttatgtga
aaagatttgaatatgagtgttgtttggtttaacag

ADF2

gttttgaccattcattcttatgtttgtttccaaagaaattgcttgtgttgaccacatttgtatagccccaacatgaagtggtaccata
tcatttaataaacacagatcatgattacactatttctaaacttgtgaacttatccaccaagttatatcacctaatccatgacattttc
aatataggttgggcaatcaaataaattcttatattatgatgattaacagaaataaaaatttaactttgggaaatagtcttatccatat
attattataataaaccaaaatgtatagaaacatataaacaaatgaaattcgtttcattcatataattcggatagcttgcaaataac
gctgacaaaaaaagaaacagtgaagaagaaaaaaaagaaggtcattttcattttctccatttgattttttgccgaccggagaaa
atatcttcttgccgtcttcttcttcatttcaccaggaacttttatttgaaatcggtttgatttagccATGgtgagctcaattttctctt
gtttctgttttaagtttttcgattttgaagctttcttctcgaaaatttcgattgcttgctagtcgatttcgagttcaaatccgtttgcttct
cttttggataacgaattctcgctcgtttagatagatctggagatgatttcagatactcgatctttggtagttgtagtgtgtgatcgga
ttagagatccgattaaagaactcgtaactttgttgttctgcttcttcgctagcttatctgaaatcaggaagaatctgctgtttttcac
ttcaagttcaaatcaagtgttaaattctacgaatttttgactttttcatcagtgtttgtcaattgttcgatctatgttgtgcttcttgcct
cttgatgtgtcaaaattgaagtcaaaatcgttctacaaagttgtgaggagctacttagcttctcttccttgtgatgttaacaatgtt
agagatgatgataatattatccaatttctgtgttaagttgtgatctttgttttagaaaatgtgtttttgaagagatgtgcatcttctca
gtgttttgttatgtgtattgatatgataacag

ADF3

agaaaatcaagtattgtttttcaaatcaagaaatatcaaagaagaattctgtattaagtttagttctcatggaagccatagaatat
atcagaagtttgaaacaaacagcaaatatatggaataaaaaacatagttattggaataaatagaatagttatttttttttgtttggc
ataaaataaagaaaattatttgttcaacaaattgttacagatttcacttatgtatattttgaactaattcttatttttatattaattaaatc
aagattttacttttgaaaaataattaatgaatttatgaaacaatctctatgtaaataaaataaagcaaattaaaacgctcacacatg
tttacttattcggactagagagcttccgcataaagctgaggaaaaaaaagagagaagacgcacaccgaagaaaacacaca
agactccattattctcctgcttcttcgtctcttcaattattttctctgagactagctttgattcaaccATGgtgagctcaagtctaa
tttttctctcttatatagttctctttctttgttctctatggcttgatttgaactttgctttctcgattcgagttccatttcatcttggattttcaa
ttcttccgatttaagttctcgcccagagttagatatgattagatctgtagatggactcgtttctttgtttagatctctgagattttgga
tttgtatggttagatggactgatctaggatatgaatcggccgatagtaaagtcttcaatttcagtgtgctttgtgttggttttagcca
attgattttgaaaccaggaagaataatagtgttccatgtggttttcctttgattttagggttacgtctatcgtttgtttagccttttcctt
ttataagatattgttttctcgcttctttgagttattatttgctgctgttgaagttttcagatgtgtgtttgattctgttgatggtagagtat
catccgattaatatgattcagaactgtaaaatctgtcaaattgatattatgatagctcaacatcatcgttcatatacgttgtggga
gtcatccacgtcttccctttgatatacacactaccaagatctgttcatgattcattatgtttgatctctgaactaatgtataacccga
acgccgttttaaaagatgggtatagtgtctactaatatgtgtattctgaagagagttgcatcttaatgttctatgttttgttggtaac
ag

ADF4

tgcgactatttcaggttcaaagtattaggaatttaggatagttccaataacgagaagagttgctcaatcgagtttggtccaataa
gataaactgaccatcaaacataaaatcctaatcgatacttttgattatttggaaacagatccaaccattggcagttgtccattgct
cattctacgcaatctttcaactgtcaaatcttagcccaacaaaaatacttagatgattattatatgttgaccaaaacaataataata
tgcttaccatatagtttattaattcataaataaattgaagaatacgaaattacgaagaaaataaataaaaatacaaaaagcaggt
tcttctctatatattcggaatttagcttccaaataacgcagaagaaagtaaagagagaagaagcaaaaagagtcatattcgttg
aaaacatcattcactcttctctgcgtctccttctttcacccgaaattgtctctgacaccgttttgattcagccATGgtgatacta
cattttcacttcttcttgttctctttttgtttatttcagttcttcaatttttaggctcgattctcgattttgctttcgtctcggattttgaata
acgattttggctcactagatctggtatatggatttgttttctccgatttagatctttgagattttgtgttgatgtgatcagagttagtg
atccacttgtgcttcgtaagttagaatcgaaatcatgaaggaatctgctaattttgtgtgtgtgctttcgaagtttcattaactattg
aacgtttctacgaccttattcgattgtttgatccatcaggcttgttgtgtgttgaagttgcaattgaagtctaaatcgttgctagag
atgtattaacgagtttatgtgtgagctattctagttttagaggtttcaggaaatttctaagattcctaaataagagattcttggctttt
agtattgtgtggattcaattataggctagctttgtgggatctgggatgtgaatcttagtgcttctgttgttttattgaaatcag

ADF6

AGAAACTGCATctctgagacacgatggcacagtcaggttccgaacgttaagagtcgccggagttggagtgaac
caccacaacagtaatcgcctagagccgtctaaaaacaaaatcaaagagattatcttaacaacgacgtcgtttcttcgttcctttt
ctacttctgaggcccaaatgatttttgttactattcactaatgggcttaattaaggcccaatattgatagatacgcggcgactagt
taagggcaacaaatatggcccacttaaaaagaaacacgacttttcttatcgcgttgttctaacgccaccgaccgaacacttc
tttttcttctgcaataaaccaaaaaaagttaaaaattcaaaaacttaattactaagcaaaagtgtgcacgaagaacaaagcgac
gcatccgattttatttgtttgataagttcgtgtcgtcttcgtctctctaacccaattctgtggtgtctgaagaaagcagaatcaaat
ccATGTCTTTCAGAGGACTTAGCAGGgtaaaattacaaaaaaaaacttttctcttcttgcttaaaatcga
tgtactcttgtctatgtcgcaagcatcttcaatgttaaaaatttgatttttatgaagtcgatgttaaaaaaaactaaacccatctcca
tttttactcttattgagggtttaagatcactagatgaagtcaattcgatgaattttggattaaaaatcgaatcttttggtatggttttg
atatgtgttttggttttatatctgtag

ADF9

Acacttaatatagttttaattggtgattttcccaattctgaacatatatatgatgataagttttccatcccaagttgtttttctcatta
gtcactggaaacattaattaccagcaatcagtagttactttggtattccccacacaaattcaaatttaaaagaaataattagtagt
acttcttatgactgtttttttactgaataaaaaagaactatatataggttgttcttaactataaccactaaaaaaaggttctaaaatt
aattttaaaaagaaaaatatatgaagttaacttttatatattacacatcaaattccaatttcaggggctggtaatttggtaataatta
aagaaactcaatggcaatgatttaagcacttctttatataagtccaccaaactacactgatctcctcaacaccacacacagcaa
aacaaaaagctctctacactcaaaatataacgaaagaacaagaagacatcatcttagctcactATGGCTTTGAAG
ACGgtaacaatatacaaactttctctatttttccacttttgatttaatgaatctaaaagaaaatattttggatgattggtatatag

Fig. S9. The prediction of ABRE core motif and GCEs in the ADFs promoter.

The prediction of ABRE core motif and GCEs in the ADFs promoter.

Fig. S10

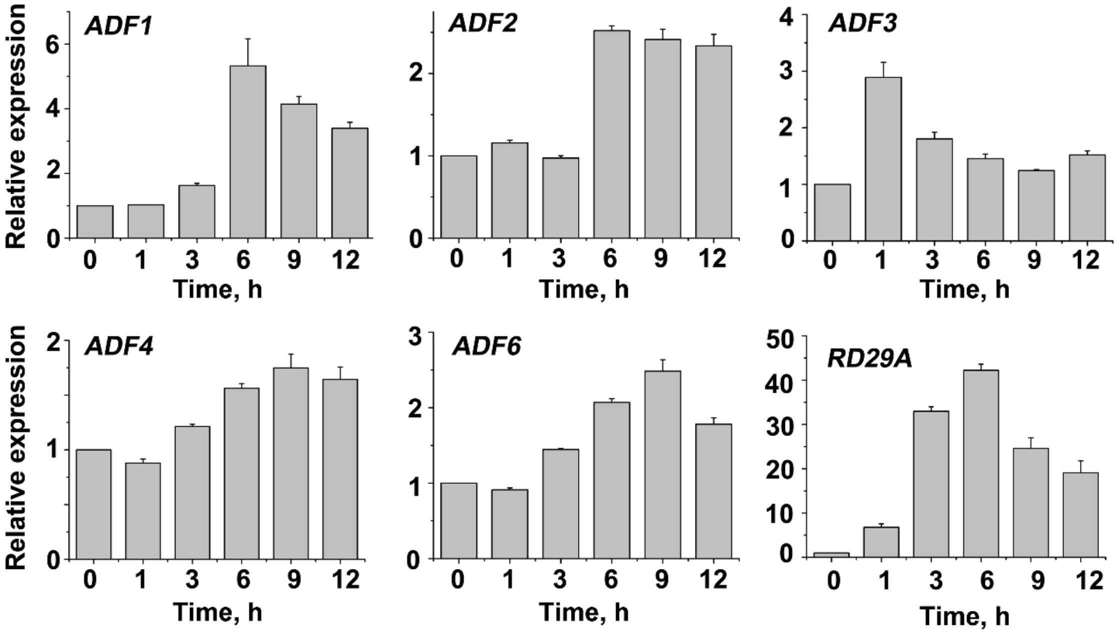

Fig. S10. The expression levels of *ADFs* induced by ABA.

qRT-PCR analysis was used to measure *ADF* (1/2/3/4/6) and *RD29A* expression by ABA. Fourteen-day-old seedlings were treated with 40 μM ABA for different times, and the leaves of seedling were detached for RNA extraction. *UBQ11* was used as an internal gene.
